# Reducing Cascading Failure Risk by Increasing Infrastructure Network Interdependence

**DOI:** 10.1038/srep44499

**Published:** 2017-03-20

**Authors:** Mert Korkali, Jason G. Veneman, Brian F. Tivnan, James P. Bagrow, Paul D. H. Hines

**Affiliations:** 1 Computational Engineering Division, Lawrence Livermore National Laboratory, Livermore, CA 94550 USA; 2 The MITRE Corporation, McLean, VA 22102 USA; 3 Vermont Complex Systems Center, The University of Vermont, Burlington, VT 05405 USA; 4 Department of Mathematics & Statistics, The University of Vermont, Burlington, VT 05405 USA; 5 Department of Electrical and Biomedical Engineering, The University of Vermont, Burlington, VT 05405 USA

## Abstract

Increased interconnection between critical infrastructure networks, such as electric power and communications systems, has important implications for infrastructure reliability and security. Others have shown that increased coupling between networks that are vulnerable to internetwork cascading failures can increase vulnerability. However, the mechanisms of cascading in these models differ from those in real systems and such models disregard new functions enabled by coupling, such as intelligent control during a cascade. This paper compares the robustness of simple topological network models to models that more accurately reflect the dynamics of cascading in a particular case of coupled infrastructures. First, we compare a topological contagion model to a power grid model. Second, we compare a percolation model of internetwork cascading to three models of interdependent power-communication systems. In both comparisons, the more detailed models suggest substantially different conclusions, relative to the simpler topological models. In all but the most extreme case, our model of a “smart” power network coupled to a communication system suggests that increased power-communication coupling decreases vulnerability, in contrast to the percolation model. Together, these results suggest that robustness can be enhanced by interconnecting networks with complementary capabilities if modes of internetwork failure propagation are constrained.

Understanding the reliability and security implications of increased coupling between interdependent power, water, transportation and communication infrastructure systems is critical, given the vital services that these infrastructures provide and continuing threats posed by natural disasters and terrorist attacks^1^,^2^. This is particularly true for the coupling between electric power and communications networks, given the essential nature of electric power to modern societies, the rapid growth of smart grid technology, and the potential for cascading failure to lead to catastrophic blackouts^3^. Smart grid technology, such as Advanced Metering Infrastructure and synchronized phasor measurement systems, leverage communication networks to enable new cyber-physical control systems designed to mitigate blackout risk^4^. But automation can also introduce new failure mechanisms: cyber-attacks may reach a larger number of critical components^5^ and outages may propagate between the coupled networks, increasing the risk of systemwide cascading failures.

Because physical experimentation with cascading failures in critical infrastructure systems is impractical, quantifying the risks and benefits of network interdependence requires the use of simulation models. A variety of models have been suggested for understanding the mechanisms by which failures, ideas, and diseases propagate within independent networks^6^,^7^. Simple models clearly show that different types of networks can respond very differently to random failures and volitional attacks^8^,^9^,^10^. Subsequently, several have suggested that topological models can provide useful insight into power grid vulnerability^11^,^12^,^13^,^14^,^15^,^16^.

However, power grids differ in important ways from these simple models. In a contagion-style model^6^,^7^,^17^, failures propagate locally: when Component *i* fails, the next component to fail is topologically connected to Component *i*. On the other hand, power grids are engineered networks, in which energy flows from generators to loads through power lines (edges), each of which has a limit on the amount of electrical flow it can tolerate. When node (substation) or edge (transmission line) outages occur, power reroutes according to Kirchhoff’s and Ohm’s laws. This rerouting increases flows along all parallel paths, which can cause a distant element of the network to become overloaded, thus initiating a chain of outages. As a result of this process, failures propagate nonlocally: the next component to fail may be hundreds of miles or tens of edges distant from the previous failure. Many temporally adjacent outages in the 1996 blackout in the Western US, for example, were spatially separated by hundreds of miles^18^. Overly simple topological models can thus lead to misleading conclusions^19^. Substantial existing research into the design of physics-based models of cascading failure in power systems^20^,^21^,^22^,^23^ suggests that one can draw useful conclusions about blackout risk, which align well with historical data, without resorting to simple topological contagion models.

This is not to say that simple models are not useful. Simple models can often suggest new approaches to a particular problem, particularly when there is limited existing understanding, as is the case with vulnerability in interdependent networks. Motivated, at least in part, by increasing interdependence between power and communications networks, a number of recent studies suggest that interdependence can increase vulnerability in network structures that were otherwise relatively robust^17^,^24^,^25^. Others have suggested that interdependent networks may be more robust to small failures, while being more vulnerable to large ones^26^. Still, others have found nonmonotonic relationships between the level of coupling between interdependent networks and network performance, suggesting that there exists an optimal level of coupling between networks^27^,^28^. Similarly, numerical experiments with a power grid model showed that there exists an optimal size for power networks^29^, suggesting that network robustness can increase by separating large, interdependent power systems. Others^30^ show that when two homogeneous flow networks are coupled together, the risk of individual network failure decreases, whereas the risk of systemwide failures increases. Finally, inspired by interdependent systems that occur in nature, researchers found that coupled networks with correlated degrees, in which hub nodes are coupled to other hub nodes, are generally robust to random failures^31^. While these results clearly show that coupling is important to the performance of interdependent networks, the “typical” impact of coupling is not clear, particularly for the case of heterogeneous networks. More work is needed to understand the conditions under which increased coupling is beneficial or harmful.

Again, most of these results come from models that diverge from real infrastructure networks in important ways, making it difficult to understand the implications for a particular system of interest. First, the topological structures found in infrastructure networks differ notably from standard abstract models^32^,^33^, largely due to geographic and cost constraints^34^. Second, the physical mechanisms of cascading within networks (see Fig. 1) and between interdependent networks^35^,^36^ differ from those of percolation^17^,^37^ and sandpile^27^ models. In order to understand the extent to which insights from abstracted network models can be useful for particular examples of interdependence (such as power and communications networks), comparisons are needed between simple models and those that capture the topology, physics, and coupling of particular infrastructure systems in more detail.

Therefore, the goal of this paper is to understand the impact of network topology, cascading mechanisms (physics), and coupling on infrastructure network vulnerability. We use the case of increased coupling between electric power systems and communication networks (a key feature of smart grid systems) as an illustrative test case. Two sets of simulation-based experiments combine to address this goal. The first set focuses on the impact of topology and physics on network robustness. In this test, we compare the relative vulnerability of different topological structures to random disturbances given two different models of intranetwork cascading: a simple contagion model and a model that more accurately captures the mechanisms of cascading in power grids. The second set of numerical experiments compares the impact of increased internetwork interdependence on vulnerability, given different models of cascading-failure propagation.

## Results

### Cascading failure in coupled power and communications networks

Because unplanned disturbances are relatively common in power grids and because large cascading failures come with enormous social costs, power systems are designed and operated so that single-element outages are highly unlikely to trigger a cascading failure. Because physical experiments are impractical, ensuring that single-element outages (known as “contingencies”) do not violate limits in a network requires the use of simulation models. Because of the importance of this problem, computing the impact of line outages on power flows was among the first applications for both analog and digital computers^38^. As a result, the technology for modeling contingencies in power systems is relatively mature.

However, multiple failures do sometimes occur, which can trigger long chains of cascading component outages. The technology for modeling these long sequences that can result when contingency analysis fails is not mature^39^. After several elements are removed from a power network, the nonlinear alternating-current (AC) power-flow equations (Supplementary Information), automated feedback control systems, and human operator actions interact in complicated ways creating many different mechanisms of cascade propagation that are often difficult to simulate. No single model perfectly captures all of these mechanisms. Models do exist that focus on particular subsets of these mechanisms such as cascading overloads^3^,^20^, hidden failures in protection systems^40^, motor stalling^41^, transient instability^18^, voltage collapse^42^, and insufficient situational awareness by operators^43^,^44^.

**Figure 1:**
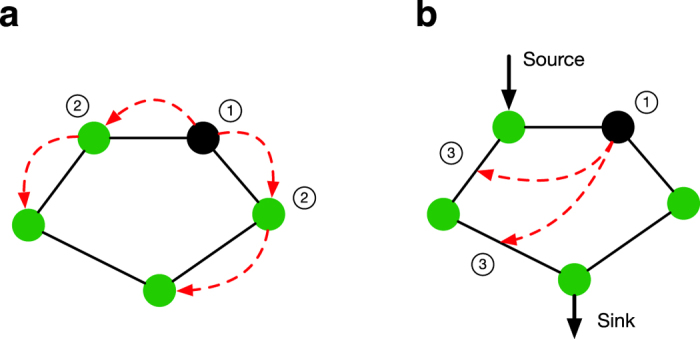
Intranetwork cascading mechanisms. Comparative illustration of cascade propagation in (**a**) topological contagion and (**b**) power grid models. In topological contagion models^6^,^27^, cascades propagate from an initiating failure① to neighboring nodes②. In a power grid, initiating failures①cause increased loads along parallel paths③, which may subsequently fail^19^.

All cascading failure models neglect some of these details, but most models, including the model used in this paper, capture the following mechanisms. When a transmission branch (line or transformer) is removed from service, the current, and thus power, flowing through that branch redistributes to parallel transmission paths throughout the network according to Kirchhoff’s and Ohm’s laws (Fig. 1). When this redistribution causes an overload, additional elements may fail. Because this redistribution progresses along all parallel paths, cascading failures often spread nonlocally. If edge (branch) outages divide the network into nonconnected components, the power injected into each connected component by generators and the power withdrawn by loads must rebalance. The first line of defense for correcting imbalances is known as the load-frequency control system, which measures local frequency and adjusts power generation to restore frequency to nominal. When load-frequency control is insufficient to correct an imbalance backup, discontinuous control systems, such as underfrequency load shedding and overfrequency generator tripping, act. Both control systems are largely decentralized, acting with locally available frequency measurements and thus do not depend on communication networks.

However, communication networks are increasingly used to enable more advanced control schemes designed to mitigate the risk and spread of cascading failures. “Automatic Generation Control” systems have long been used to actively correct regional supply and demand imbalances. In some regions, “Special Protection Schemes”^45^ are used to automatically trigger remote stress-mitigating control actions (such as disconnecting noncritical loads) if critical contingencies occur. And communication systems increasingly enable human operators to quickly combine real-time measurements and computer models to quickly choose and actuate stress-mitigating systemwide control actions.

None of these schemes depends on the public internet. Instead, proprietary SCADA (supervisory control and data acquisition) networks connect many, but not all, high-voltage nodes in a power network over custom-built communication networks that typically combine fiber-optic, microwave, and telephone communication channels. Most nodes in SCADA communication networks use battery-based power supplies, providing some assurance that power outages will not also result in communication outages. Because SCADA networks have coevolved with the physical infrastructure and are inherently tied to geography, their topological features are often highly correlated with those of the physical power infrastructure.

However, public communications systems do have some role in power system operations. For example, communication failures were reported to impede efforts to restore the Italian power system after the cascading failure of September 28, 2003^46^, thus increasing the temporal duration of the blackout. But this is different from increasing the geographic scope of the cascade, as implied by simple coupled topological models^17^,^24^,^47^,^48^.

**Figure 2:**
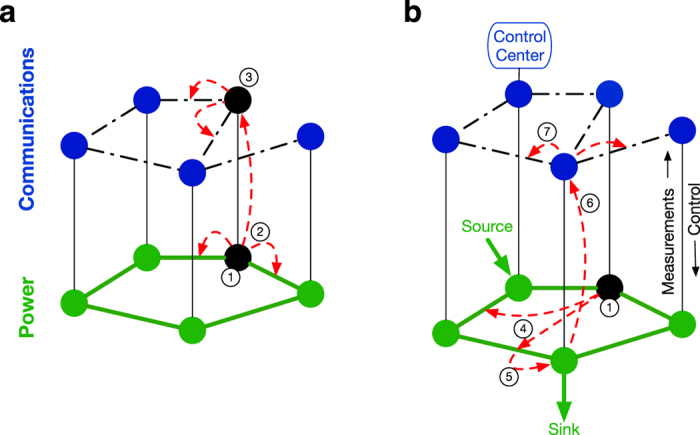
Internetwork cascading mechanisms. Comparative illustration of the (**a**) “Coupled Topological” model^17^ in which failures propagate immediately from the power network to the communications network and the (**b**) “Intermediate” Smart Grid model where failures can propagate within the power network and have a chance of causing communications failures. In the Coupled Topological model, an initiating disturbance ① causes ② edge failures in the power grid as well as ③ node and edge failures in the communications (comm) network. As a result, the size of the giant component is reduced to 0.8*N*. In our smart grid models, the initiating failure① potentially causes overloads③, which causes an edge failure and #x02464; a loss of power at the “sink” node. This may (depending on the availability of backup power) cause a communication node failure ⑥ and thus communication link failures ⑦, which fracture the communication network and prevent messages from being passed from and to the control center.

In order to capture these key characteristics of historical cascading failures, our model (DCSIMSEP/C) has the following structure. First, the power flowing along each transmission line is computed using a power-flow model. This initial model was designed to ensure that all power flows begin at or below their rated limits. Second, initiating outages are chosen at random (in this paper, we simulate node or bus outages) and applied to the network. Third, if outages result in the division of the network into multiple nonconnected components, each component is rebalanced using a combination of increasing or decreasing generator output (up to 5% of their rated limits) and, if this is insufficient, a combination of load shedding and generator tripping. Fourth, new state variables are computed to satisfy power-flow equations (Supplementary Information). Fifth, measurements are gathered from and control actions are applied to the power network (Fig. 2). This step relies critically on the health and connectivity of the communication network. Sixth, the most overloaded element is removed from the network, simulating the actions of relays and circuit breakers. This process then repeats from step three until power flows are below their limits, or the simulation time exceeds some threshold, which, in this case, is 30 minutes of simulated time.

Understanding how interdependence will impact cascading-failure sizes requires an understanding of what mechanisms of internetwork cascade propagation exist. The link from power to communications clearly comes from the fact that SCADA nodes require energy from the grid to operate. However, SCADA nodes (typically known as “Remote Terminal Units”) almost universally use battery backup systems to reduce the likelihood of failure propagation from the power grid to the communication network. However, there remains some nonzero chance that these backup systems will fail to operate when they are needed. In the other direction, when a communication node fails, power nodes do not immediately fail. Instead SCADA node failures make it impossible for human operators and centralized automated control systems to monitor and control a particular power node; leaving that particular location to operate based on power grid physics alone. In addition, SCADA node failures may separate the communications network into nonconnected components, thus preventing operators from interacting with particular components of the power network.

Since the precise nature of power grid-communications interdependence depends on many factors that vary from one location to another, this paper considers three possibilities for the nature of the power-communications coupling. In all three of our models in which the communications network is used (our “Smart Grid” models), cascades are allowed to propagate within the power grid as described above, but the communication network now has the ability to collect measurements and issue control commands to the grid with the aim of mitigating cascades. Each model begins with two graphs: a power network, 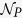, and a communication network, 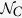, and a set of connections between the two. If there is an 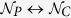 connection at Node *i* and a contiguous path from Node *i* to the network’s centrally located control center through 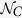, then the control system is able to collect measurements from Node *i*, such as flow data from adjacent transmission lines and network topology changes. Similarly, sources or sinks at Node *i* can be remotely controlled only if there is a path from Node *i* through 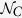 to the control center. Without this connection, generators and loads react to imbalances in supply and demand in ways that mimic existing decentralized control systems. Given a valid connection to the control center, measurements can be collected and used to choose optimal control actions (i.e., rapid reductions in nodal supply or demand) that could mitigate propagation of the cascade (see Methods). Once chosen, control decisions are distributed through 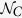 to the appropriate nodes in 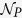. Choosing optimal control actions in this way mimics the behavior of power system operators who are constantly using network models to choose stress-mitigating control actions when unexpected stresses arise. In most cases, wide-area emergency controls rely on the actions of human operators, but automated approaches to wide-area control are increasingly common in the literature^49^ and in industry practice^50^. Thus DCSIMSEP/C captures the essence of the ways in which centrally located operators (human or cyber-physical) react to unexpected stresses in a power system.

**Figure 3:**
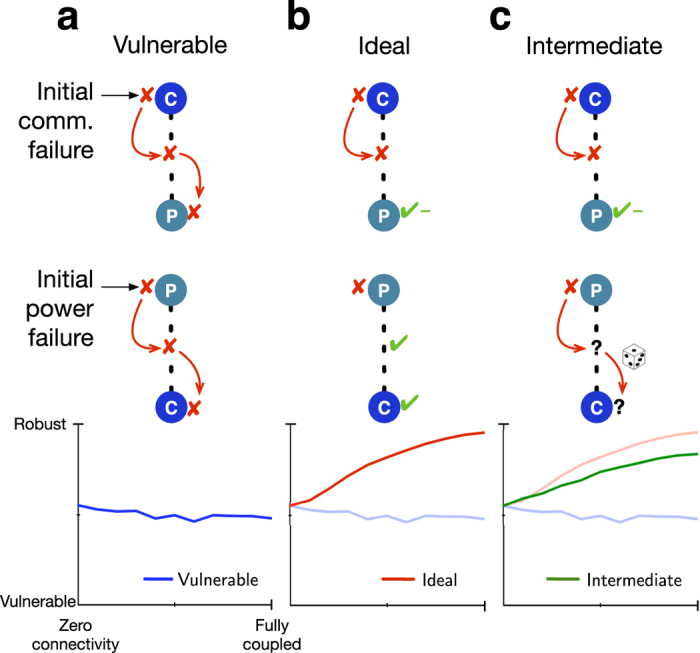
Illustration of the three smart grid models. In the Vulnerable model (**a**), communication failures cause grid failures, and power failures cause communication failures, both with probability one. As the connectivity between the power grid and the communication network increases, the robustness of the network decreases. In the Ideal model (**b**), communication failures can degrade the ability to monitor and control power nodes, but power failures cannot return to cause additional communication failures. In this case, as connectivity increases, robustness increases. In the Intermediate model (**c**), communication failures degrade control performance as before, and power failures trigger communication failures probabilistically. Robustness is degraded, but still increases monotonically with connectivity.

Our first variant on this model (“Vulnerable”) has generators and loads at Node *i* that fail (trip) immediately when the corresponding communication Node *i* fails and there is an 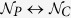 connection at Node *i*. This is the most pessimistic of the three models, and diverges substantially from industry design standards that aim to minimize the likelihood of a power failure causing communication failures, and vice versa. Since the possibility exists for node failures in 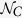, nodes in 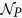 will lose the ability to be monitored and controlled if there ceases to be a functional communication path between the control center and a particular grid node. If communication node/edge failures cause 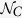 to fracture into multiple connected components, signals can only pass within the component where the control center is located (Fig. 2). Figure 3a illustrates this[Fig f4][Fig f5] behavior and shows results (detail in Fig. 6) suggesting that increased power-communications coupling can make power grids more fragile to random disturbances.

The second variant (“Ideal”, Fig. 3b) is the opposite. It assumes that communication nodes continue to operate, even if nodes in 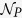 fail. This corresponds to the case where the SCADA network has high-reliability battery backup systems that allow it to continue to operate irregardless of failures in the power network. In the Ideal model, increased coupling results in monotonically increasing robustness.

The third variant (“Intermediate”, Fig. 3c) models a plausible midpoint between these two extremes. In this version, communication nodes fail with a probability that is proportional to the fraction of local load shedding that has occurred at that node. For example, if Node *i* in 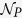 has lost 50% of its local load, Node *i* in 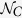 will fail with probability 0.5, which may cut off communication routes to/from the control center. This reflects the fact that transmission substations typically have backup power systems (typically batteries), but most have a limited amount of onsite storage. As outages become more severe, backup systems will be increasingly taxed, increasing the likelihood that power will be lost at these locations. The Intermediate model reflects this situation by scaling the probability that power failures reach the communication network with the size of the local power failure. As shown in Fig. 3c, this model shows reduced robustness relative to the Ideal model, but robustness still increases monotonically with increased coupling.

### Networks and metrics

The remainder of this section presents results from two sets of simulation-based experiments aimed to understand how different network topologies respond to random node failures of various sizes, under several different models of cascading. Five different topological structures were simulated: a square lattice, an Erdős-Rényi random graph, a random regular network, a scale-free network, and a model of the Polish power grid^51^. Each of these initial networks was sized to have *N* = 2,383 nodes and *M* = 2,886 links to correspond with the size of the Polish grid model.

In each experiment, we vary the size of the initiating failure, *f*, which is the ratio of the number of nodes in the initial random failure to the total number of nodes in the network, *N*. The ultimate impact of each initiating failure is measured by finding the size of the largest (giant) connected component of the graph after the cascade has subsided, *N*_∞_, or (for the power grid models) the amount of demand served at the conclusion of the cascade, *D*_∞_. For each *f* we report, as our metric of robustness, the probability that more than half of the original *N* nodes remain within the giant component at the conclusion of the cascade: *P*_*N*/2_ = Pr(*N*_∞_ > 0.5 *N*). For the power network models, we measure the probability that at least half of the original demand is served at the conclusion of the cascade, which we denote by *P*_*D*/2_ = Pr(*D*_∞_ > 0.5 *D*). In this paper, we use the term vulnerability to mean the opposite of robustness (e.g., 1 − *P*_*N*/2_). In some cases, we compare the vulnerability of network-structure/cascade-model combinations from the area under the *P*_*N*/2_ vs. *f* curve (Supplementary Information).

### Intranetwork cascading

Our first set of numerical experiments compares the robustness of five different network structures to random node failures using the two different models of cascade propagation illustrated in Fig. 1: a simple topological contagion model and a (not-communication-enabled) power grid model.

**Figure 4 f4:**
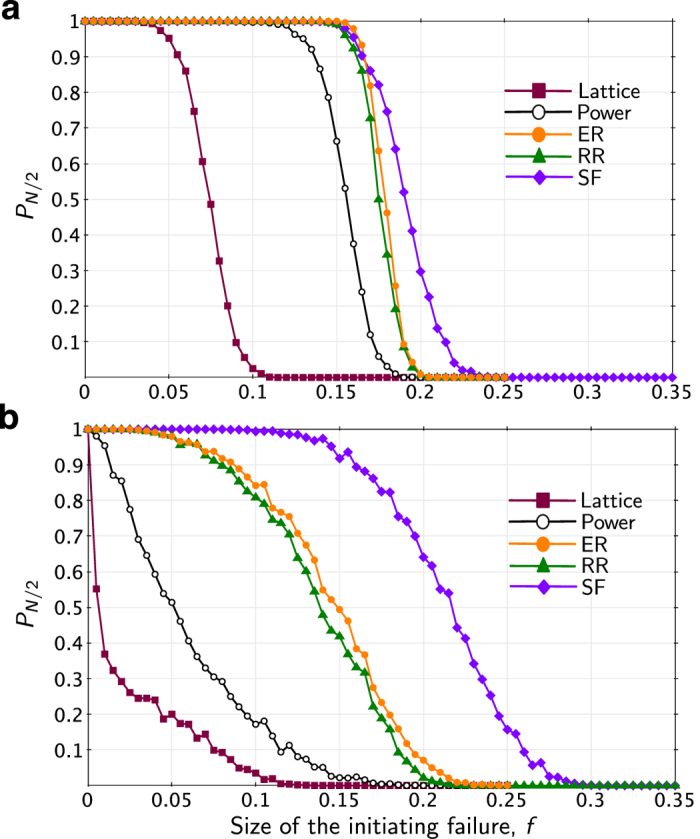
Robustness (*P*_*N*/2_) of several network structures to random node failures. Panel (**a**) shows results from a model of topological contagion and (**b**) shows results from a model of cascading in power systems.

Figure 4a shows the topological contagion results, using a model proposed by Watts^6^. In this model, after the initial set of *fN* node failures, Node *i* fails if the fraction of Node *i*’s neighbors that are in a failed state exceeds a threshold *ϕ*_*i*_, which was randomly drawn for each node *i* from a uniform distribution over (0, 1). Figure 4b shows results from the power grid model. For these simulations, we rewired the transmission lines from the original Polish power network according to the appropriate synthetic network type. While real power networks clearly deviate from the synthetic topologies, this rewiring allows us to understand the impact of network structure on network performance.

The results from these two models show some notable similarities. From both models of cascading, the power grid and lattice structures appear to be most vulnerable and the scale-free topology is the most robust. In fact, the relative order of the five networks is nearly identical in Fig. 4a,b.

On the other hand, the Power Grid model accentuates the robustness differences among the different topologies and changes the nature of the transition in *f*. In the Power Grid model, we do not observe the rapid, second-order phase transition that is apparent in the topological model; transitions as *f* increases are more gradual. This may result from averaging over many abrupt transitions as the number of initiating failures increases, as was previously reported for transitions in vulnerability with increasing load levels^23^. Whereas the midpoint of the transition is similar in the two models (i.e., Power Grid and Topological Contagion) for the scale-free network, the Polish power grid and lattice structures appear to be much more vulnerable from the perspective of the Power Grid model.

### Internetwork cascading

Our second set of numerical experiments explores the impact of increased coupling between networks on network vulnerability. Specifically, we considered a pair of interdependent networks (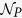 and 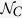), in which a fraction *q* (degree of coupling) of the *N* nodes in 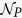 are coupled to corresponding nodes in 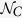. As in the first set of simulations, two different types of models are compared: one that is purely topological (Fig. 2a) and a second that follows the cascading-failure model introduced previously (Fig. 2b).

The first model is an implementation of the interdependent cascade/percolation model proposed by Buldyrev *et al*.^17^. In this paper, we start with two coupled networks, 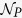 and 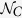, each of which has sufficient internal connectivity to form a single connected component. Random initiating failures were applied to nodes on network 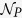, and the incident edges on that network immediately fail and are removed. If the removed edges result in a connected component in 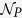 (or 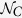) that includes a different set of nodes from those in the coupled network, then the edges linking the components in 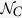 (or 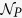) fail. This cascading process continues until both 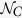 and 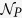 have the same set of connected components. Hereinafter, this model will be referred to as the “Coupled Topological Model”.

Because of the fact that SCADA networks are typically custom-built to monitor and control a particular power grid, the topology of a SCADA network is typically strongly geographically correlated with that of the infrastructure system to which it is coupled. Thus, 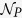 and 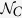 are likely to be somewhat, but not perfectly, correlated. To approximate this correlation, 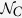 was initialized to be identical to 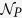, and then 10% of the edges in 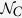 were randomly rewired (see Methods).

After initializing the data and models, the various models were, as before, subjected to random node failures, and the performance of the networks was measured. For the Coupled Topological results, we measured network performance using the giant component probability, *P*_*N*/2_. For the Smart Grid models, we measured both *P*_*N*/2_ and an analogous measure of performance: the probability that the network can serve at least 50% of the load in the network, after the cascade has subsided, *P*_*D*/2_ (see Methods).

**Figure 5 f5:**
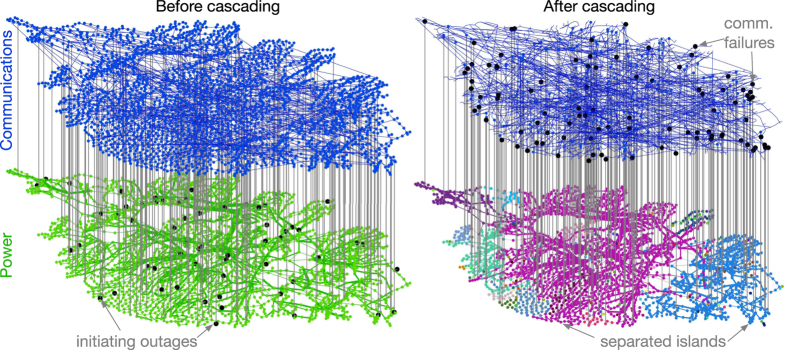
The coupled power-communications model, applied to the Polish power system test case. The left panel shows the topology of the power and communications networks, along with the initiating node (bus) outages and the internetwork links. The right panel shows the locations of line outages that subsequently occurred in the “Intermediate” Smart Grid model. Colors in the power network on the right show the separation of the grid into nonconnected components as a result of cascading.

Figure 5 portrays the initial state of the coupled system (the left panel) with 10% coupling (i.e., *q* = 0.1), together with the state of the two networks after the cascade has terminated (the right panel) in the Intermediate Smart Grid model, wherein the 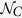-nodes fail with a probability that is proportional to the fraction of load shedding that has occurred at the corresponding 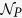-nodes.

**Figure 6:**
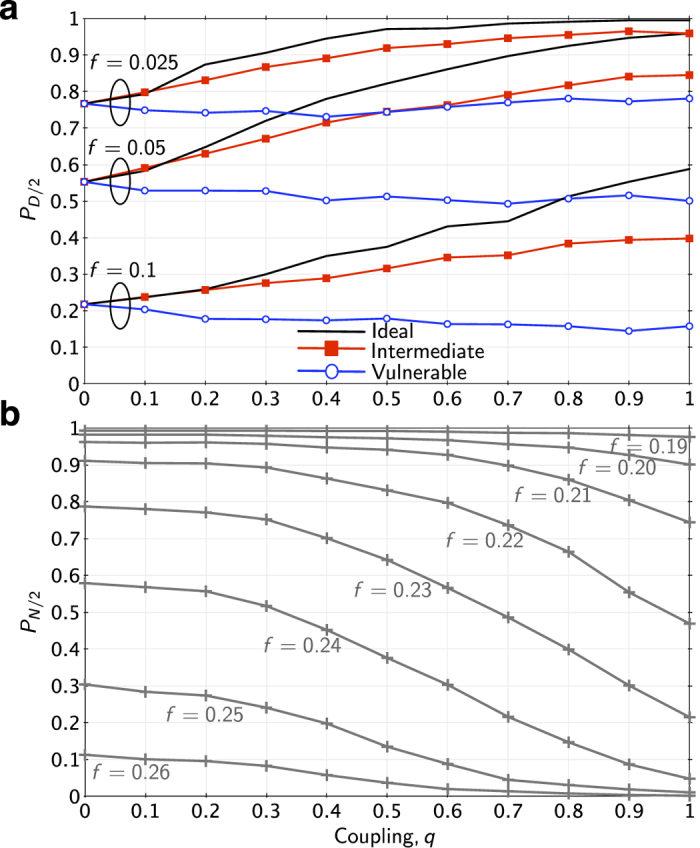
Robustness of the Polish network to random failures, with varying levels of coupling, *q*. Panel (**a**) shows results from four different models of cascading in power grids, three of which are coupled to communications systems, after 5% of nodes initially failed (*f* = 0.05). In this case, we measured robustness with the probability that at least half of the original demand is served at the conclusion of the cascade. Panel (**b**) reports analogous results from the Coupled Topological model, for several different failure sizes, with robustness measured as in Fig. 4.

Figure 6 shows the results for fixed failure sizes, *f* = 0.05, and varying levels of coupling, *q*. For *q* = 0 (i.e., uncoupled networks), the smart grid models produce results that are identical to the uncontrolled power grid, since cascading occurs only within the power grid; the communication network neither benefits nor detriments the system. As *q* increases, the robustness of the Ideal and Intermediate models increases monotonically. For the Vulnerable Smart Grid model, robustness decreases monotonically with *q*. In contrast, for the Coupled Topological model, robustness decreases monotonically with *q*; the “optimal” level of coupling is *q* = 0 for all initiating failure sizes, *f*. It is interesting to note that the results from both types of model contrast with prior results^27^ that suggest that there exists an optimal level of coupling between *q* = 0 and *q* = 1. In all of these cases, optimal performance results are obtained at either *q* = 0 or *q* = 1.

In order to compare the Intermediate model to the Coupled Topological model in more detail for different types of topological structures, we took the four additional network topologies from Fig. 4, and connected them to correlated communication networks, using the same method used with the Polish power network. Both models, with full coupling, *q* = 1, were subsequently subjected to random node failures as before, measuring the robustness of the networks to different disturbance sizes (varying *f*).

**Figure 7:**
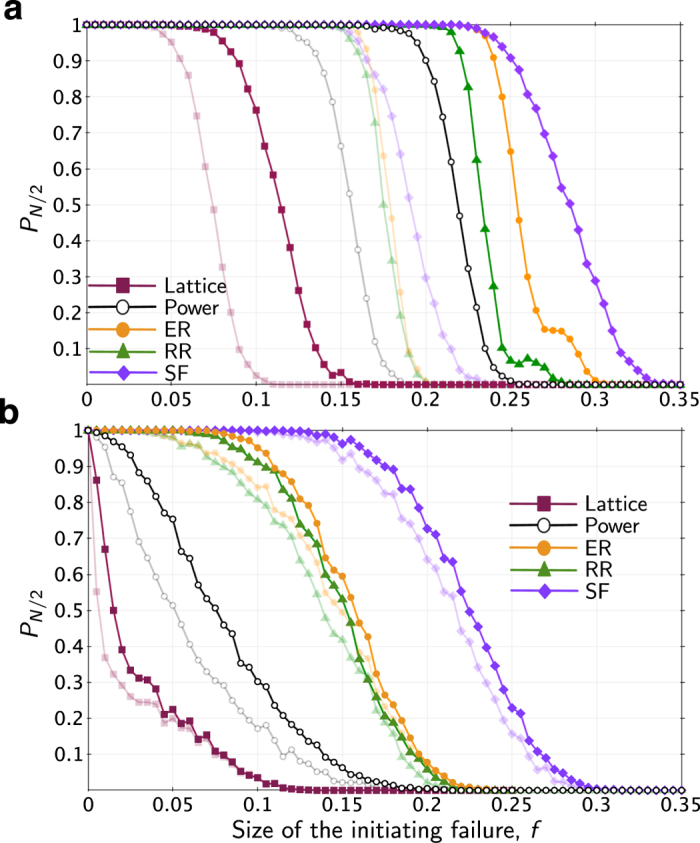
Robustness of fully coupled networks, *q* = 1, to random failures. In the (**a**) Coupled Topological cascading model and in the (**b**) “Intermediate” Smart Grid model. The shadowed data are duplicated from Fig. 4a,b in order to compare the coupled and single-network models.

Figure 7 shows the results. Comparing these results to the single-network contagion results in Fig. 4 suggests that coupling networks together always increases vulnerability, regardless of topology. However, for the power grid model, a similar comparison of the Intermediate Smart Grid model to the baseline Power Grid model (Fig. 4) suggests that increased interdependence always increases robustness, regardless of topology.

## Discussion

Together, these results have important implications both for the emerging science of interdependent networks and for the design of intelligent, cyber-physical infrastructure systems.

Firstly, the power grid and topological models show several important qualitative similarities. The relative vulnerability of the different network structures to random failures is similar across the various models studied in this paper. Lattices are consistently the most vulnerable and scale-free networks are consistently the most robust; power grids perform only slightly better than lattice topologies. This indicates that topological structure does have an important impact on the vulnerability of power networks, and that some aspects of this impact are captured in simple topological models of cascading.

However, this is where the similarities end. When we measured the effect of network coupling on performance, increased coupling consistently increased network robustness in all but the most extreme (and unrealistic) Smart grid model. For the Ideal and Intermediate models, the most robust configuration was the fully coupled case, *q* = 1. In the Coupled Topological model, *q* = 0 was the optimal level of coupling^28^, and robustness monotonically decreased with increased coupling. For every attack size and every topological structure, increased coupling increased vulnerability in the Coupled Topological model and decreased vulnerability in the more realistic smart grid models. The reason that vulnerability decreased in the smart grid models is that interconnections between the two networks performed valuable functions in arresting the spread of cascades. When components were overloaded, and thus at risk of cascading, the communication network facilitated valuable systemwide control functions. Since the communication network’s beneficial functions are not modeled in the Coupled Topological model, increased coupling decreases robustness. However, in real infrastructure systems, as in biological systems^31^, networks are typically coupled together because coupling enables some synergistic function. These differences indicate that models of network interdependence can lead to misleading conclusions if those models do not adequately capture the beneficial functions of coupling in addition to describing the various mechanisms by which cascades can increase propagation between coupled systems.

Clearly, these results come from numerical simulations that include important assumptions about how power systems react to stress and how cascades propagate within and between coupled networks. Understanding exactly what mechanisms of cascading in power systems need to be modeled in order to obtain statistically accurate results is an ongoing challenge^39^. The actual behavior of a particular power system coupled to a particular communication network will certainly differ from the simulations in this paper. However, these results clearly show that conditions exist under which the coupling of one network to another can improve the performance of the coupled systems. Similarly, these results show that when evaluating the performance of coupled systems, one needs to understand not only the detrimental impacts of coupling, but also the beneficial functions that can come from increased interconnectivity. Understanding both the benefits and risks of interconnections is key to the design of robust, resilient systems in a world in which infrastructure networks are inextricably interdependent.

These results suggest several practical design practices for interdependent infrastructure systems. In the case of the Ideal and Intermediate Grid models, increased coupling was more beneficial than detrimental because of the limited ways in which cascades could propagate between the two networks. In practice, limits on internetwork cascades can be implemented by sound engineering practices that reduce the chance of failures propagating between networks. For example, adding reliable, well-maintained backup power systems to critical components is an effective strategy for reducing harmful interdependence. In some cases, such as the use of backup battery systems for SCADA communications, this strategy has already been implemented with substantial success. However, other harmful infrastructure interdependencies for which there are low-cost solutions persist. For example, adding battery backup systems to traffic signals along critical transportation corridors is a relatively low-cost way to reduce coupling between power and transportation networks^52^.

## Methods

### Network topological data

In this study, five different topological structures were studied. Power network data came from a model of the Polish power grid that is publicly available with MATPOWER^51^. This model has *N* = 2,383 nodes (buses) and *M* = 2,886 edges (transmission lines or transformers), after removing parallel edges. For comparison, four synthetic networks were generated according to the standard Erdős-Rényi (ER)^53^, random regular (RR)^54^, preferential attachment (scale-free, SF)^55^, and square-lattice attachment kernels^56^. In order to ensure that the synthetic graphs had the same size as the power network, we randomly removed edges from the initial topological configurations as needed to produce graphs with the correct size. Edge removals that would result in the graph separating into nonconnected subgraphs were avoided in order to ensure that the predisturbance graphs were fully connected. Similarly, duplicate edges and self-loops were removed for consistency between the synthetic graphs and the power grid data.

### Generating synthetic power grid data

After building graphs that were identical in size to the 2,383-node Polish power grid, we generated synthetic power grid data for each of the synthetic graphs. In order to locate sources and sinks within the synthetic networks, each of the generators and loads in the Polish network was randomly assigned to one node in each network. In addition, each Edge (transmission line) *i* ↔ *j* was given a normalized impedance of 1, such that the normalized power flowing from Node *i* to Node *j*, after the linearized direct-current (DC) power-flow assumptions (Supplementary Note), was *F*_*ij*_ = *θ*_*i*_ − *θ*_*j*_, where *θ*_*x*_ is the phase angle of the sinusoidal voltage at Node *x*. Flow limits on each transmission line were determined by taking the flow limits from the original Polish network data and randomly assigning each limit to one of the links in the synthetic network. After this was done, the line limits were increased as needed to ensure that no single-line outage would result in a cascading failure, as is common practice in power systems.

### Generating communications network topologies

Geographically correlated communication network, 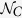, data were generated as follows. First, we made a copy of the corresponding power network such that 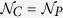. Then, we randomly rewired 10% of the edges in 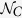, excluding rewirings that would result in self-loops or duplicate edges. Then, nodes in the two parallel networks were interconnected. Specifically, Node *i* in 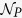 was connected to Node *i* in 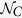 with probability *q* ∈ [0, 1]. The resulting interlinks produce a correlated pair of graphs (as illustrated in Fig. 2), which are at least somewhat similar to the correlated topologies found in real power and communication networks. Once each 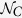 was formed, we located a “control center” at the node in 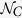 with the highest betweenness centrality.

### Modeling cascading failures in power grids

Our model of cascading failure in power systems (DCSIMSEP/C) extends prior work on cascade modeling by the authors^57^ and others^3^,^58^,^59^, which are closely related to random fuse networks^60^. In this model, power flows are computed using the DC power-flow equations (Supplementary Information). The DC model can be summarized as follows:


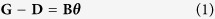



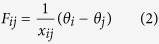


where **G** and **D** are vectors of power generation and load; **B** is a weighted Laplacian matrix that encodes the network’s topology; ***θ*** is a vector of voltage phase angles; *F*_*ij*_ is the power flow from Node *i* to Node *j*; and *x*_*ij*_ is the (normalized) inductance of the transmission line. When a component fails, flows are recomputed according to equations (1) and (2). If the revised power flows exceed the flow capacity, this line will open (disconnect) in an amount of time that is proportional to the overload. This changes the configuration of the network (changing **B**), causing the flows to be recomputed. If the network separates into islands, there may not exist a feasible solution to equation (1) due to an imbalance between supply and demand. To correct this imbalance, a combination of generator adjustments and load reductions are used to arrive at a new, feasible solution of equation (1).

### Smart grid models

The three smart grid models each depend on an optimization problem that identifies control actions (load shedding and generator reductions) in order to mitigate overloads on transmission lines. This problem seeks to minimize the amount of load shedding and power generation reductions necessary to arrive at a feasible solution to equations (1) and (2), with the added (soft) constraint that each flow *F*_*ij*_ should be within the flow capacity limits for this link. The optimization proceeds as follows. After each 1 minute of simulation time, the centrally located control center collects measurement data (power flows, line status (open/closed), generator states and load states) from all of the nodes for which there exists a connected path between the control center and that node. Let 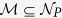 represent this set of measurable nodes and edges, 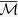 represent the unmeasurable nodes, 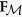 represent the vector of measured power flows, 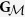 represent the vector of measured generator states, and 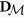 represent the vector of measured load states. For the Ideal Smart Grid model, 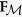, 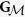, and 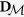 are always full vectors of all measurements from nodes that have communication network connectivity (given *q*). Depending on the level of coupling, *q*, and the state of the communication network, 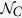, these may be subvectors of all possible measurements. After the control center gathers measurements 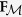 through the communication system, it solves the following optimization problem:


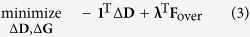



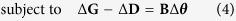



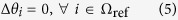



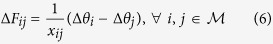



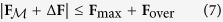



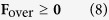



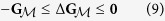



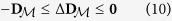



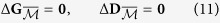


The objective for this problem (3) is to minimize the total amount of load shedding (−**1**^T^**ΔD**) plus the weighted sum of all overloads that cannot be eliminated through changes to generators and loads (**λ**^T^**F**_over_). For this work, we set **λ** to be uniform weight vector such that each λ_*i*_ = 100 (in normalized units); however, we found that the results are largely insensitive to this parameter (see Supplementary Fig. S6).

Constraint (4) enforces that the net changes to nodal power injections (**ΔG** − **ΔD**) must be equal to the changes in power flowing out through transmission lines (**BΔ*****θ***). Constraint (5) fixes one voltage phase angle, *θ*, in each connected component of the network as a reference; Ω_ref_ represents this set of reference nodes. Constraint (6) computes the changes in flow on each of the measured transmission lines. Equation (7) attempts to limit the post-optimization power flows (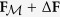) to be below the flow limits, **F**_max_. The vector **F**_over_ in (3) and (7) turns the flow constraint into a soft constraint, which alleviates the problem of occasionally infeasible cases, particularly when the system is very heavily stressed. **F**_over_ is constrained to be nonnegative in inequality (8). Constraints (9) and (10) ensure that the system exclusively reduces load and generation at measured nodes (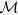) in its attempt to eliminate overloads on transmission lines. Finally, equation (11) forces the system to not change load or generator at nodes that are not accessible from the control center (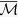). These assumptions are similar to prior work by Parandehgheibi *et al*.^36^.

Each of the three smart grid models makes use of this optimization problem in a slightly different way. The Ideal Smart Grid model uses perfect information about all communication-connected nodes to solve this problem, optimally choosing adjustments to the available generators and loads, independent of where they are in the network. If there is no communication link to a particular node, the Ideal Smart Grid model does not gather data about flows from this location, and assumes that it has no ability to control generators or loads at this node. Thus, the topology of 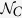 does not impact the Ideal model.

The Intermediate model, however, does rely on the state of the communication network. The optimizer can only control and monitor nodes when there is an 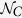-path between a particular grid node and the control center node. When the path to Node *i* is broken, the optimization formulation is adapted to exclude generation and load at Node *i* from the set of control variables, and it ignores the flow constraints adjacent to Node *i* (e.g., the flow constraint on Edge *i* → *j*), unless an adjacent node (e.g., *j*) is connected to the control center. In addition, the Intermediate model assumes that if there is load shedding at grid Node *i*, the adjacent communication node will fail with probability that is equal to the fraction of load shedding.

The Vulnerable Smart Grid Model adds to this the rather extreme assumption that if a communication node fails, the generation and load at that node will also fail.

### Measuring the initiating failure size

Note that our measure of attack size, *f*, as shown in Figs 4, 6 and 7, is the complement of the notation used by Buldyrev *et al*.^17^. In our notation, *f* represents the size of the initiating attack (or random failure). In Buldyrev *et al*.^17^, *p* = 1 − *f* represents the fraction of the *N* nodes in each network that remain in service immediately after an initial, random set of *f* = ∼(1 − *p)N* node failures. *f* was used, rather than *p*, for clarity of presentation, particularly for readers who are less familiar with the percolation literature.

### Measuring robustness, vulnerability, and sample size

Our measure of robustness, *P*_*GC*_ = *P*_*N*/2_, differs slightly from the traditional *P*_∞_-measure, which is commonly used in the percolation literature and which averages GC sizes across a set of samples. Since power networks are small, relative to (for example) thermodynamic systems, the underlying rationale for *P*_∞_ is less robust. In our judgement, the *P*_*GC*_-measure more clearly presented the results. However, we computed results using both metrics and found that the *P*_∞_-measure led one to the same conclusions as reported in this paper. See Supplementary Information for a comparison of the results with *P*_GC_ and *P*_∞_.

In this paper, each estimate of *P*_GC_ comes from the simulation of 1,000 random initiating disturbances of size *f* and counting the number of cases that result in a cascade with the end-state largest connected component containing at least 0.5 *N* nodes. This sample size (1,000) was found to provide a reasonable balance between variance in *P*_GC_ and computational requirements, which were substantial given the more detailed nature of our models. To compute the variance, we used standard bootstrapping methods and found the standard deviation of *P*_GC_ to be almost universally less than 0.01.

In discussing the vulnerability of the various models we frequently suggest, based on the sigmoidal *f*-*P*_GC_ curves, that one network/model combination is more or less vulnerable than another. Many different measures, such as stochastic dominance or the point at which *P*_GC_ drops below 0.5, could be used to reach nearly identical qualitative comparisons; however, our primary metric for comparison is the area under the *f*-*P*_GC_ curve, which is larger for networks that are more robust to random failures. The inverse of this is thus a measure of vulnerability (Supplementary Information).

### Data and materials availability

Computer code for the models and analysis methods described in this paper, and other information can be found online at https://github.com/mitre-rise/coupled-networks.

## Additional Information

**How to cite this article:** Korkali, M. *et al*. Reducing Cascading Failure Risk by Increasing Infrastructure Network Interdependence. *Sci. Rep.* **7**, 44499; doi: 10.1038/srep44499 (2017).

**Publisher's note:** Springer Nature remains neutral with regard to jurisdictional claims in published maps and institutional affiliations.

## Supplementary Material

Supplementary Information
