# Erratum: Reducing Cascading Failure Risk by Increasing Infrastructure Network Interdependence

**DOI:** 10.1038/srep46959

**Published:** 2018-03-19

**Authors:** Mert Korkali, Jason G. Veneman, Brian F. Tivnan, James P. Bagrow, Paul D. H. Hines

Correction to: Scientific Reports 7: Article number: 4449910.1038/srep44499; published online: 03 20 2017; updated: 03 19 2018

The original HTML version of this Article contained typographical errors in the legend of Figure 2.

“In our smart grid models, the initiating failure ① potentially causes overloads ③ which causes an edge failure and #x02464; a loss of power at the “sink” node.”

now reads:

“In our smart grid models, the initiating failure ① potentially causes overloads ④ which causes an edge failure and ⑤ a loss of power at the “sink” node.”

