# Supplementary material for: Reducing Cascading Failure Risk by Increasing Infrastructure Network Interdependence
Source: Sci Rep. 2017 Mar 20;7:44499. doi: 10.1038/srep44499 (PMC5357958; doi:10.1038/srep44499)
Supplement: Supplementary Information [file srep44499-s1.pdf]

Supplementary Information for "Reducing Cascading Failure Risk by Increasing Infrastructure Network Interdependence"

Mert Korkali, Jason G. Veneman, Brian F. Tivnan, James P. Bagrow, and Paul D.H. Hines

Supplementary Note

DC power-flow model

In this paper, we made use of the "DC power flow" linearization of the full nonlinear power flow equations in our model of cascading failures. Here, we briefly describe the derivation of this common, although imperfect, simplification. For a more detailed discussion of the DC power-flow equations and their limitations, see refs S1, S2.

Consider a node ("bus" in power systems terminology) k that is connected to node m via a transmission line, which has series resistance r_{km} and reactance $x_{km} = \omega l_{km}$, where ω is the frequency of the sinusoidal current and l_{km} is the series inductance of the line. r and x can be combined to form a complex impedance $z_{km} = r_{km} + jx_{km}$, in which (by electrical engineering notational tradition) $j = \sqrt{-1}$. The inverse of this impedance is known as an "admittance," and is defined as follows: $1/z_{km} = y_{km} = g_{km} + jb_{km}$, where g and b are known, respectively, as the conductance and susceptance of the line. The sinusoidal voltages at nodes k and m will each have an amplitude (V) and a phase shift (θ, relative to some reference), and can thus be represented with complex numbers $\tilde{V}_k = V_k e^{j\theta_k}$ and $\tilde{V}_m = V_m e^{j\theta_m}$. Note that voltages V_k, and consequently all other units, are normalized to dimensionless units, using the 'per-unit' system, such that 1.0 indicates a nominal value. With these definitions, we can define the complex current $\tilde{I}$ and power $\tilde{\mathbb{S}}$ flowing out from k to m as:

$$\tilde{I}_{km} = y_{km}(\tilde{V}_k - \tilde{V}_m) \tag{S1}$$

$$\tilde{\mathbb{S}}_{km} = \tilde{V}_k I^*_{km} = \tilde{V}_k(\tilde{V}^*_k - \tilde{V}^*_m)y^*_{km} \tag{S2}$$

where x^* indicates the complex conjugate of x. With some manipulation of equations (S1) and (S2), we can find the active ($\mathscr{P}$) and reactive ($\mathbb{Q}$) power flowing from k to m as follows:

$$\mathscr{P}_{km} = V_k^2 g_{km} - V_k V_m (g_{km} \cos\theta_{km} + b_{km} \sin\theta_{km}) \tag{S3}$$

$$\mathbb{Q}_{km} = -V_k^2 b_{km} - V_k V_m (g_{km} \sin\theta_{km} - b_{km} \cos\theta_{km}) \tag{S4}$$

where $\theta_{km} = \theta_k - \theta_m$ is the phase angle difference between k and m. If we assume that the voltage amplitudes V_k and V_m are at their nominal levels, that we have normalized y_{km} such that this nominal level is 1.0 (common practice), and that the resistance r_{km} is small (nearly zero) relative to the reactance x_{km} (a reasonable assumption for bulk power systems), then $g_{km} \cong 0$, and $\mathscr{P}_{km}$ becomes:

$$\mathscr{P}_{km} \cong -b_{km} \sin\theta_{km} = \frac{1}{x_{km}} \sin\theta_{km} \tag{S5}$$

If we assume that θ_{km} is small, then $\sin\theta_{km} \cong \theta_{km}$ and we get:

$$\mathscr{P}_{km} \cong \frac{1}{x_{km}} \theta_{km} \tag{S6}$$

If we furthermore assume that $\mathbb{Q}_{km} = 0$ (not a particularly good assumption), then the current magnitude and the power are equal, $|I_{km}| = \mathscr{P}_{km}$, and we can use equation (S6) to roughly simulate power flows in a power system.

In order to solve for the flows $\mathscr{P}_{km}$ in simulation, we put equation (S6) into matrix form as follows. Let $\mathbf{A}$ denote the line-to-node incidence matrix with 1 and -1 in each row indicating the endpoints of each line, θ be the vector of voltage phase angles, $\mathbf{X}$ be a diagonal matrix of line reactances, and $\boldsymbol{\mathscr{P}}_{\text{flow}}$ be a vector of active power flows along

transmission lines. Then, we can solve for the vector of power flows $\mathscr{P}_{\text{flow}}$ given that we know the vector of voltage phase angles θ as shown in the following:

$$\mathbf{A}^{\top}\theta = \mathbf{X}\mathscr{P}_{\text{flow}} \tag{S7}$$

$$\mathscr{P}_{\text{flow}} = \left[\mathbf{X}^{-1}\mathbf{A}^{\top}\right]\theta \tag{S8}$$

In order to solve for θ, we use information about the sources (generators) and sinks (loads) to build a vector of net injected powers (generation minus load), $\mathscr{P}$. Given $\mathscr{P}$, we can solve the following to find θ:

$$\mathscr{P} = \mathbf{A}\mathscr{P}_{\text{flow}} = \left[\mathbf{A}\mathbf{X}^{-1}\mathbf{A}^{\top}\right]\theta = \mathbf{B}\theta \tag{S9}$$

The matrix $\mathbf{B}$ is known as the bus susceptance matrix, and has the properties of a weighted graph Laplacian matrix describing the network of transmission lines, where the link weights are the susceptances $b_{km} = 1/x_{km}$.

Comparing $P_{N/2}$ to P_∞

In this paper, we measured the impact of disturbances of various sizes, f, on the probability of at least half of the network remaining within the "giant component" (GC) after the resulting cascade had subsided: $P_{N/2}$; or the probability of half of the load still being served after the cascade completed: $P_{D/2}$. An alternative way to measure the impact of the disturbances is to measure the average cascade size (sometimes known as the *yield*), rather than the probability of a cascade in a given size range. Indeed, after random removal of fN nodes from the network, $N_0 = (1-f)N$ nodes remain in the GC, and the cascade of failures results in gradual fragmentation of the network, leading to a reduction in the number of nodes in the GC until a post-cascade steady state is reached. At this point, we calculated the ratio of the number of nodes remaining within the end-state GC, N_∞, to the size of the network after the initial removal of nodes, N_0, and measured the average cascade size, $\langle N_\infty/N_0\rangle$, across a set of samples. This measure would be more analogous to the P_∞-metric that is commonly used in the literature on phase transitions in percolation systems. We chose not to use P_∞ as our primary measure of network robustness since the modeling assumptions described in the above discussion of "DC power flow" become particularly inaccurate for very large cascades. Essentially, P_∞ would, in many cases, average over small numbers that were not particularly accurate.

However, the results that one obtains by measuring the average cascade impact do not lead one to substantially different conclusions than those reported in the paper (aside from the fact that the transitions are much more gradual).

Supplementary Figure S1 compares the response of various networks to random failures using the P_∞- and $P_{N/2}$-measures for the topological contagion and power grid models. For the power grid model, the relative robustness of the five network structures is unchanged. The lattice is the most vulnerable and the scale-free network is the most robust. In the topological model, the P_∞-measure indicates that the power grid, random graph, random regular, and scale-free networks have similar levels of robustness, for $f < 0.15$. The lattice remains to be the most vulnerable of the five network structures.

Supplementary Figure S2 compares the response of various coupled models to random failures with different levels of coupling between the power and communications network. In this case, we compare the original metrics used in the paper (i.e., $P_{N/2}$ and $P_{D/2}$) to P_∞. Our analogous measure of robustness for the three smart grid models is $\langle D_\infty/D\rangle$: the average ratio of the amount of demand (load) served at the end of the cascade to the original load, which amounts to 24.5725 GW. The results for the three different smart grid models are not substantially changed. We still see that increased coupling increases robustness in both the Ideal and the Intermediate Smart Grid models, whereas coupling is detrimental (though only slightly) in the Vulnerable Smart Grid model. For the Coupled Topological model, coupling is detrimental to robustness; indeed, by measuring the results using both P_∞ and $P_{N/2}$, the decrease in performance with q is monotonic.

50% coupling results

To better understand the impact of the level of coupling, we recomputed the results shown in Supplementary Fig. S4 using 50% coupling, i.e., $q = 0.5$.

Network vulnerability indices

One way to compare the various topological configurations and models described in this paper is to convert the sigmoidal results shown in Supplementary Figs S3 and S4 into a single metric of robustness (or conversely, vulnerability). To quantify the effects of topology, physics, and coupling among different synthetic networks, we define the *network vulnerability index* (β) as follows:

$$\beta = -\log \int_0^1 P_{\mathrm{GC}}(f)\,\mathrm{d}f \tag{S10}$$

$$\approx -\log \left\{ \frac{1}{2L} \sum_{\ell=1}^{L-1} P_{\mathrm{GC}}(f_\ell) + P_{\mathrm{GC}}(f_{\ell+1}) \right\} \tag{S11}$$

where f is the initiating failure size; L is the total number of f values simulated; and $P_{\mathrm{GC}} = P_{N/2}$ is the probability of observing a GC whose size is more than half the number of grid nodes. The β-values corresponding to five network structures and six models of cascading studied in this paper are aggregately displayed in a bar chart in Supplementary Fig. S5.

Sensitivity analysis on λ

Sensitivity of the three smart grid models with respect to the weight vector λ using various levels of coupling ranging from $q = 0$ to $q = 1$ is displayed in Supplementary Fig. S6. This analysis is carried out using our load-based P_∞-measure, in which the ratios of the amount of post-cascade power, D_∞, to the precascade load amount, D, are averaged across the same 1,000 initial outage sets of failure size $f = 0.05$, as in other cases. It is clearly seen that the choice of the weight vector, λ, of unavoidable overloads has only negligible impact on the values of the chosen robustness metric, $\langle D_\infty/D \rangle$.

Supplementary References

S1. Stott, B., Jardim, J. & Alsac, O. DC power flow revisited. *IEEE Trans. Power Syst.* **24**, 1290–1300 (2009).

S2. Gómez-Expósito, A., Conejo, A. J. & Cañizares, C. (eds.) *Electric Energy Systems: Analysis and Operation* (CRC Press, Baco Raton, FL, 2009).

Supplementary Figures

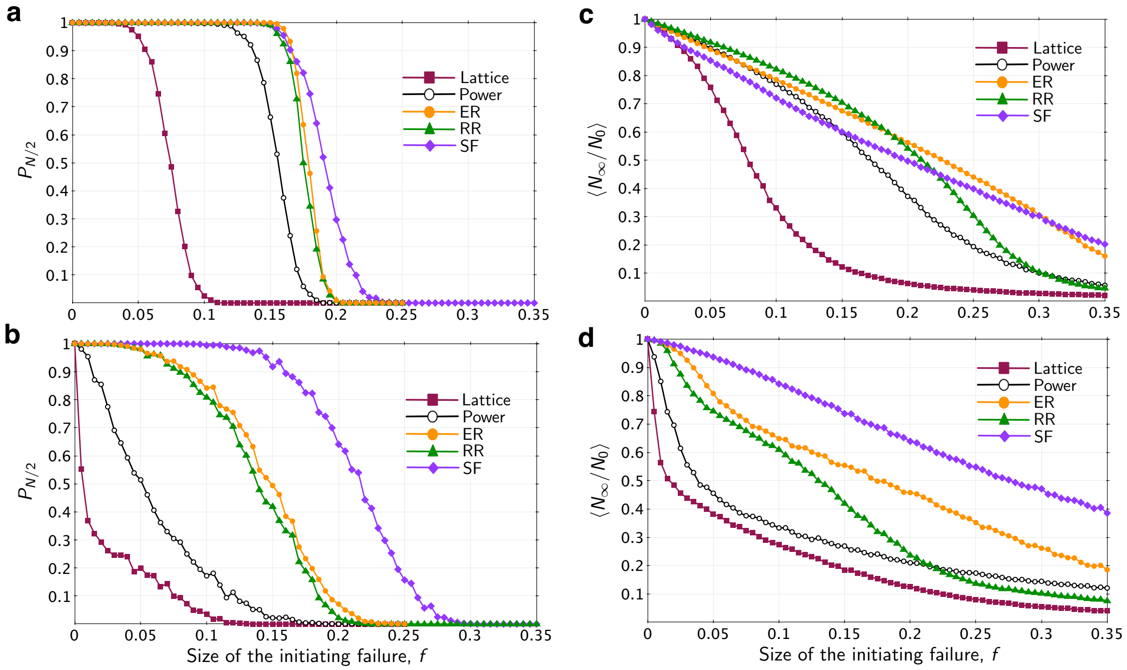

Supplementary Figure S1. The response of cascading models to random node failures. Simple model of topological contagion (**a**,**c**) and our power grid model (**b**,**d**). (**a**,**b**) show the response using the $P_{N/2}$-measure used in the paper. (**c**,**d**) show the response using the average size of the post-cascade giant component, $\langle N_\infty/N_0 \rangle$.

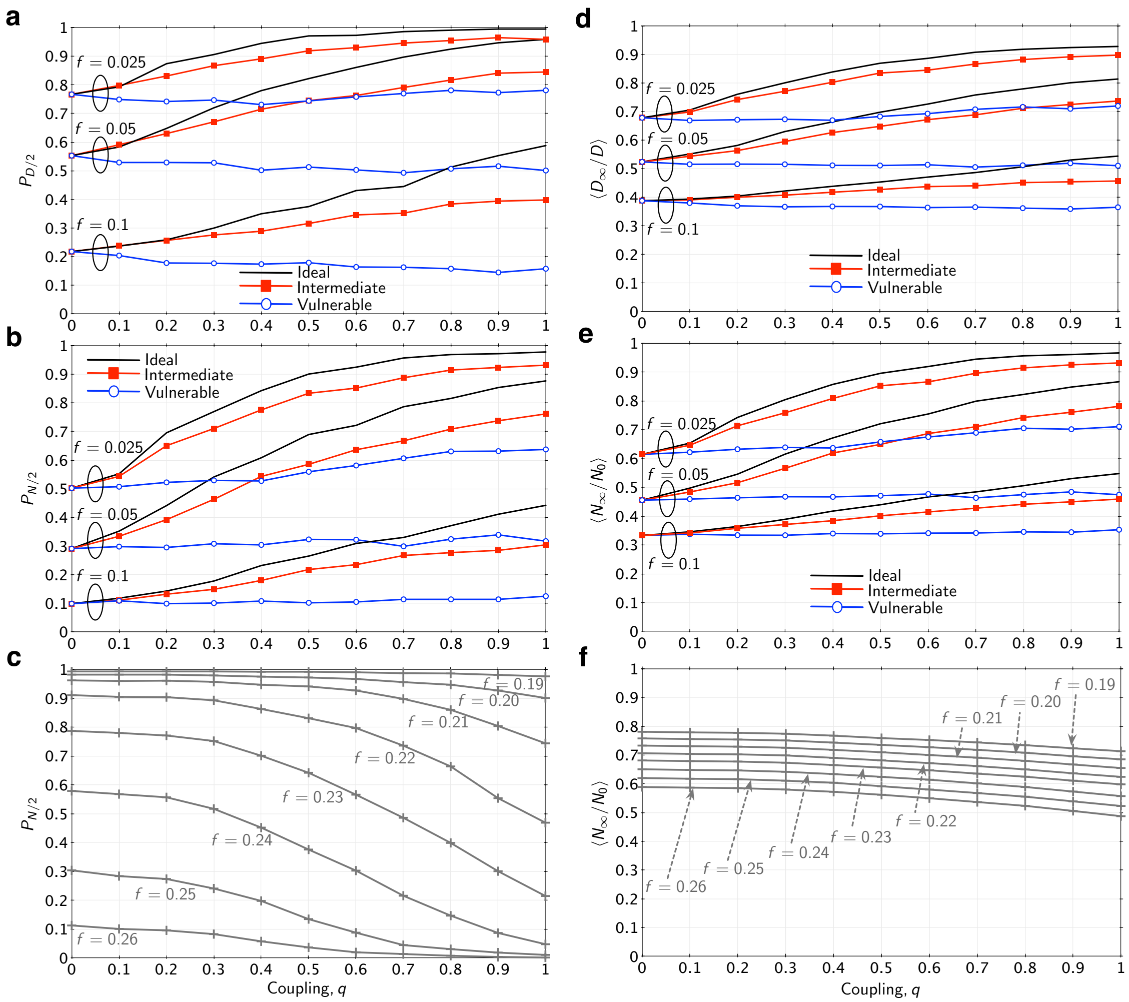

Supplementary Figure S2. The robustness of the Polish power grid topology, when coupled to a communication network, for two different models and two different measures of robustness, as a function of the level of internetwork coupling, q. (**a**,**b**,**d**,**e**) show results for the four power grid models, whereas (**c**,**f**) show the Coupled Topological model. (**a–c**) measure robustness using the $P_{D/2}$- and $P_{N/2}$-measures, as in the main paper, whereas (**d–f**) use measures that are more analogous to P_∞.

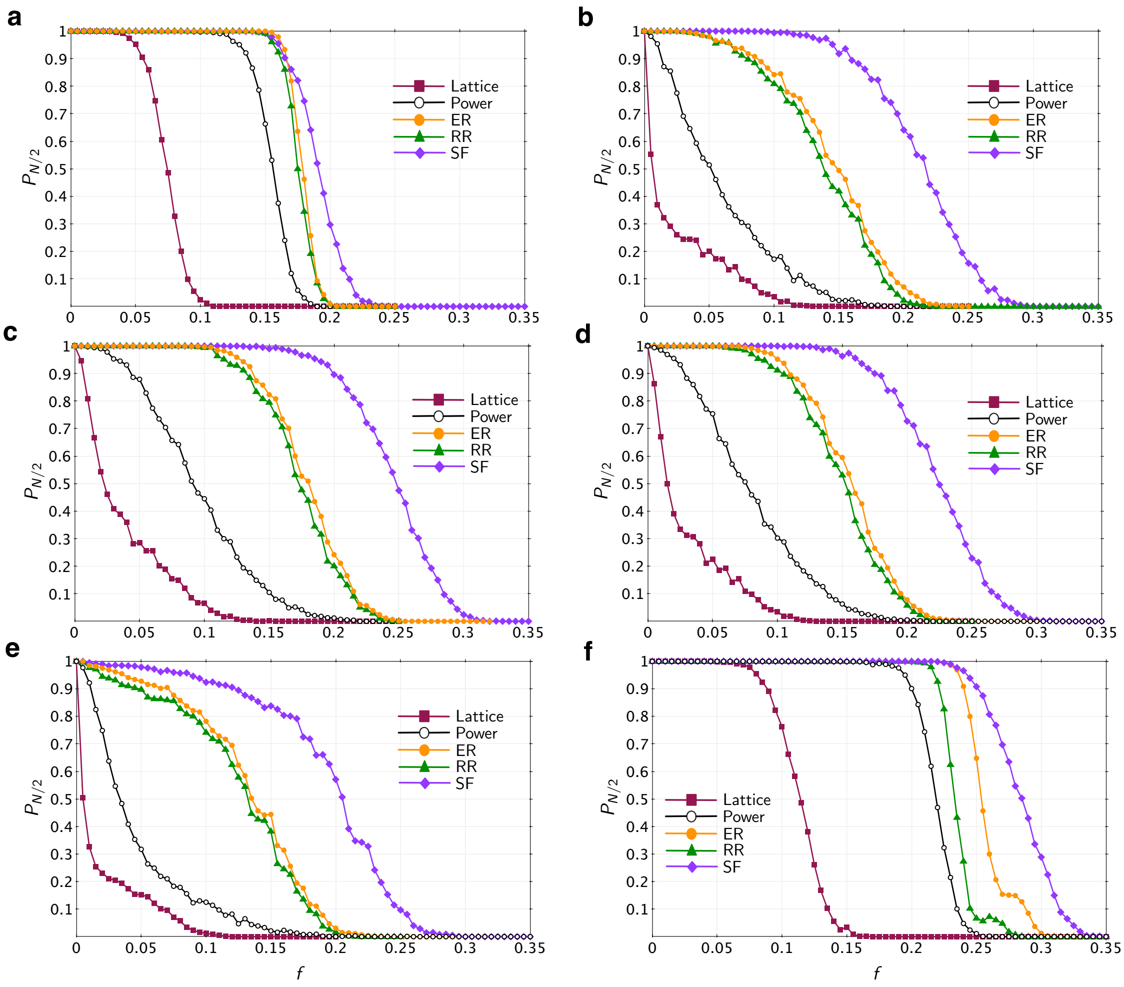

Supplementary Figure S3. Comparison of the robustness for all six of the models considered in this paper. (**a**) Single-network, topological cascading, (**b**) single-network, power-flow-based cascading, (**c**) Ideal, (**d**) Intermediate, (**e**) Vulnerable, and (**f**) Coupled Topological cascading model. In all of the coupled models, the grid and communication nodes are assumed to be *perfectly* coupled, i.e., $q = 1$.

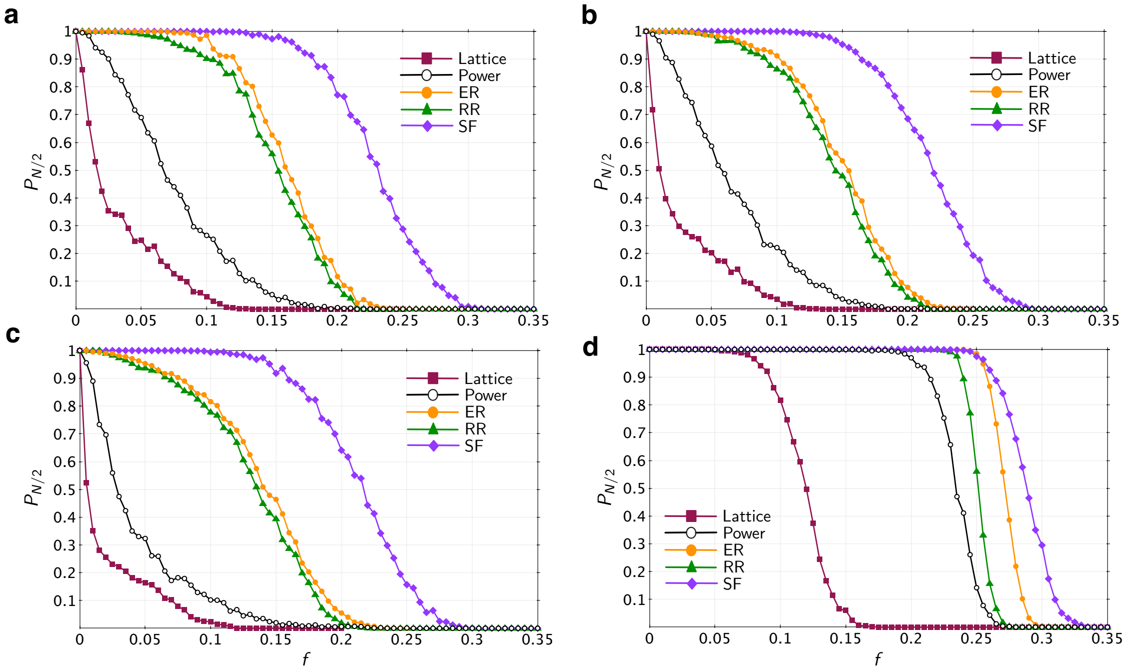

Supplementary Figure S4. Comparison of robustness results for the four coupled network models, with 50% coupling, $q = 0.5$. The models are the (**a**) Ideal, (**b**) Intermediate, (**c**) Vulnerable, and (**d**) Coupled Topological cascading model.

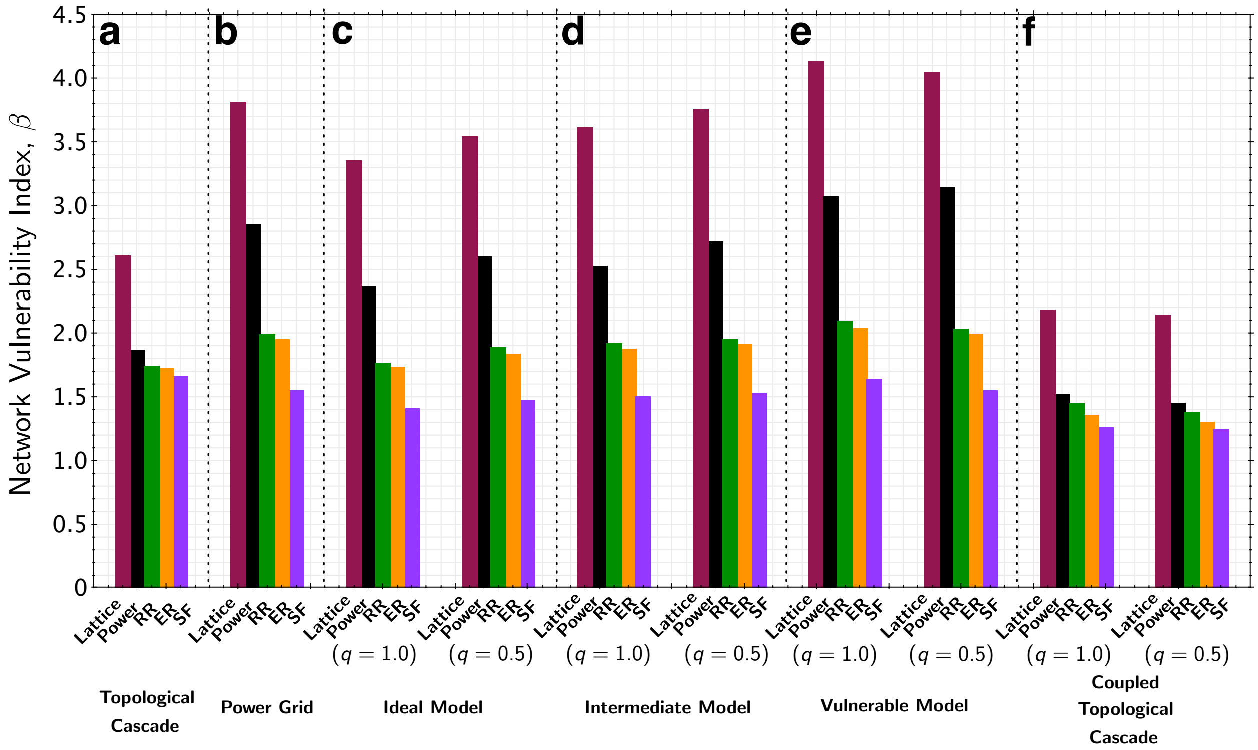

Supplementary Figure S5. Network vulnerability indices, β, for different models of cascading for the fully coupled ($q = 1.0$) and half-coupled ($q = 0.5$) cases. (**a**) shows the simple topological cascading model; (**b**) shows the uncoupled power grid; (**c–e**) show the three smart grid models; and (**f**) shows the Coupled Topological model.

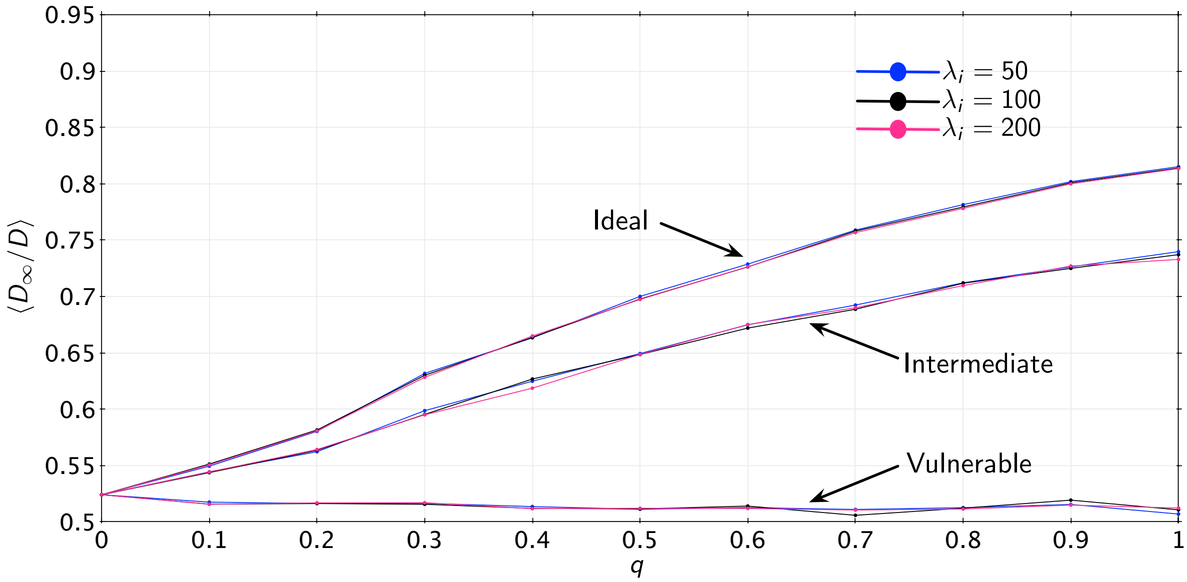

Supplementary Figure S6. Sensitivity of the three Smart Grid models to the overload weight vector, λ, for overloads that cannot be eliminated through changes to generators and loads for the Polish power grid. The $\langle D_\infty/D \rangle$-results shown herein are produced using three different uniform weight vectors (i.e., with $\lambda_i = 50, 100, 200$), varying levels of coupling from $q = 0$ to $q = 1$, and a failure size of $f = 0.05$.
